# Outcome, demography and resource utilization in ICU Patients with delirium and malignancy

**DOI:** 10.1038/s41598-021-98200-8

**Published:** 2021-09-21

**Authors:** Mattia Sieber, Alain Rudiger, Reto Schüpbach, Bernard Krüger, Maria Schubert, Dominique Bettex

**Affiliations:** 1 Department of Internal Medicine, Zuger Kantonsspital, Landhausstrasse 11, 6340 Baar, Switzerland; 2 Department of Medicine, Spital Limmattal, Urdorferstrasse 100, 8952 Schlieren, Switzerland; 3 Institute of Intensive Care, University Hospital Zurich and University of Zurich, Rämistrasse 100, 8091 Zurich, Switzerland; 4 Cardio-Surgical Intensive Care Unit, Institute of Anesthesiology, University Hospital Zurich and University of Zurich, Rämistrasse 100, 8091 Zurich, Switzerland; 5 School of Health Professions, Institute of Nursing, Zurich University of Applied Science, Technikumstr. 81, P.O. Box, 8401 Winterthur, Switzerland

**Keywords:** Medical research, Oncology

## Abstract

Delirium in the general intensive care unit (ICU) population is common, associated with adverse outcomes and well studied. However, knowledge on delirium in the increasing number of ICU patients with malignancy is scarce. The aim was to assess the frequency of delirium and its impact on resource utilizations and outcomes in ICU patients with malignancy. This retrospective, single-center longitudinal cohort study included all patients with malignancy admitted to ICUs of a University Hospital during one year. Delirium was diagnosed by an Intensive Care Delirium Screening Checklist (ICDSC) score ≥ 4. Of 488 ICU patients with malignancy, 176/488 (36%) developed delirium. Delirious patients were older (66 [55–72] vs. 61 [51–69] years, *p* = 0.001), had higher SAPS II (41 [27–68] vs. 24 [17–32], *p* < 0.001) and more frequently sepsis (26/176 [15%] vs. 6/312 [1.9%], *p* < 0.001) and/or shock (30/176 [6.1%] vs. 6/312 [1.9%], *p* < 0.001). In multivariate analysis, delirium was independently associated with lower discharge home (OR [95% CI] 0.37 [0.24–0.57], *p* < 0.001), longer ICU (HR [95% CI] 0.30 [0.23–0.37], *p* < 0.001) and hospital length of stay (HR [95% CI] 0.62 [0.50–0.77], *p* < 0.001), longer mechanical ventilation (HR [95% CI] 0.40 [0.28–0.57], *p* < 0.001), higher ICU nursing workload (B [95% CI] 1.92 [1.67–2.21], *p* < 0.001) and ICU (B [95% CI] 2.08 [1.81–2.38], *p* < 0.001) and total costs (B [95% CI] 1.44 [1.30–1.60], *p* < 0.001). However, delirium was not independently associated with in-hospital mortality (OR [95% CI] 2.26 [0.93–5.54], *p* = 0.074). In conclusion, delirium was a frequent complication in ICU patients with malignancy independently associated with high resource utilizations, however, it was not independently associated with in-hospital mortality.

## Introduction

Delirium is a common acute brain dysfunction in patients hospitalized in the intensive care unit (ICU). It is characterized by a sudden onset and fluctuating course of inattention, alteration of consciousness and cognitive impairment^1,2^. The frequency ranges from 19% in postoperative patients to 82% in severely ill mechanically ventilated patients^1,3–5^. It has been demonstrated that delirium is associated with a prolonged ICU and hospital length of stay (LOS), more ventilator days, higher costs, increased in-hospital and long-term mortality as well as long-term cognitive impairment^1,6–8^. While most findings on delirium in the ICU originate from general ICU populations, delirium in oncological patients has mainly been investigated in general wards and palliative care units. In these settings, cancer patients show a high delirium frequency with higher rates in palliative care units, a pronounced morbidity as well as an increased hospital and post-discharge mortality^9^.

However, the frequency of delirium and the associated impact on the outcomes of ICU patients with malignancy have not yet been thoroughly investigated. These topics have been evaluated only by two studies both of which had small sample sizes, were underpowered and reached contradictory results with respect to delirium as a predictor of mortality^10,11^. Better understanding the role of delirium in those patients’ ICU stay and outcome is important for several reasons: (1) ICU patients with malignancy may be at high risk of developing delirium since they represent a seriously ill population whose underlying malignancy, its potential complications as well as medications used in the management are associated with increased risk of delirium^12^; (2) ICU patients with malignancy are an important ICU subpopulation as their number has been increasing in the last two decades and may continue to do so^13^; (3) More knowledge on this population may have relevant implications for clinical routine and health care costs.

The aim of the present study was to assess the frequency of delirium in critically ill oncological patients and to investigate the associated patient characteristics and impact on resource utilizations and outcomes. To address these questions, we assessed delirium in all patients admitted to different specialized ICUs across one university hospital during one year. We subsequently performed a subgroup comparison between delirious and non-delirious oncological patients with respect to patient characteristics, resource utilization and outcomes.

## Methods

### Study design

This retrospective, single-center longitudinal cohort study at a University Hospital in Switzerland was part of a large Health Service Research project, which evaluated the prevention, screening and treatment of delirium in hospitalized patients. Results from the overall cohort including 10,906 hospitalized patients have been reported recently^14^.

### Setting

This University Hospital has approximately 39,000 admissions annually distributed across 43 departments and institutes. In the year 2014, a total of 4002 patients were treated in one of the specialized ICUs for medical, abdominal and thoracic surgical, cardiovascular surgical, trauma surgical, and neurosurgical as well as burn patients.

In the year 2012, a concept for delirium management and Health Service Research project (Delir Path) was launched in all departments by a multi-disciplinary and multi-professional expert team. By covering all aspects from screening to pharmacological and non-pharmacological treatment its aim was the improvement of prevention, early recognition and treatment of delirium in hospitalized patients. Physicians and nurses received training via lectures, e-learning modules and bedside teaching, and had access to the developed algorithms available as pocket cards and on the hospital’s intranet. These algorithms comprised the screening of all patients with a Richmond Agitation Sedation Scale (RASS)^15^ score of − 3 to + 4 with the Intensive Care Delirium Screening Checklist (ICDSC)^16^, performed by trained nurses once per shift. Positive delirium screening corresponding to an ICDSC score ≥ 4 was followed by appropriate pharmacological treatments and non-pharmacological measures. The drug of first choice was the neuroleptic pipamperone, which exists as tablets or as syrup and is strongly sedative while having a weak anti-psychotic action. If hallucinations occurred the neuroleptic drug haloperidol was added orally or intravenously. Vegetative symptoms were treated with intravenous clonidine or dexmedetomidine. Patients with nocturnal agitation, insomnia or increased risk of non-convulsive epileptic seizures received intravenous midazolam via a continuous infusion with doses between 0.05–0.1 mg/kg/h with daily infusion interruptions at 6:00 a.m. in order to screen for delirium.

### Participants

Data of all adult patients ≥ 18 years admitted to one of the six ICUs between 1st of January and 31st of December 2014 were included in the longitudinal cohort study. Patients from intermediate care units and patients with missing data and/or ICDSC score were excluded from the analysis. Patients screened with the Confusion Assessment Method for the ICU (CAM-ICU) instead of ICDSC were also excluded due to previous data from our study center suggesting higher sensitivity with ICDSC^17^. However, patients with pre-existing neurologic conditions were not excluded.

### Definitions of delirium and malignancy

Patients were considered delirious if the ICDSC score was ≥ 4. The ICDSC is one of the most widely used screening methods in the ICU setting and comprises eight criteria assessed over one entire nursing shift. Initial validation, meta-analysis and previous studies by our center showed that the chosen ICDSC cut-off score of ≥ 4 has good sensitivity (62–99%) and specificity (57–95%) as well as moderate to good reliability (κ 0.59–0.92)^16–19^. However, the different criteria of screening tests do not equally contribute to the test’s diagnostic performance^20,21^.

Patients were categorized as having malignancy when the principal diagnosis had been attributed to an ICD-10 code from the International Classification of Diseases (ICD) which belongs to the block C (“malignant neoplasms”) from chapter II (“neoplasms”).

### Outcome variables

Outcome variables of interest were in-hospital mortality, ICU and hospital LOS in hours and days, respectively, duration of mechanical ventilation in hours, ICU nursing workload assessed with the nine equivalents Nursing Manpower Use Score (NEMS)^22^, costs in the ICU and total costs per case in Swiss Francs, assessed by the hospital and provided to the Swiss Federal Statistical Office, as well as the rate of patients discharged home.

### Potential confounders

The Charlson Comorbidity Index was calculated according to Quan et al.^23^, with higher values signifying a higher comorbidity burden. The Simplified Acute Physiology Score II (SAPS II) indicating disease severity was computed with the worst values during the first 24 h of the ICU stay^24^. Sepsis and shock were determined using the ICD-10 codes from principal and secondary diagnoses.

### Data sources

All data are documented in the patient medical records. They refer to the Swiss Federal Statistical Office^25^ medical and administrative database and the database Minimal Data Set—Intensive Care Unit (MDSi)^26^. Authorized administrative personnel extracted the data of interest and provided it to the investigators. The researchers had no possibility to identify patients from whom data were collected.

### Statistical analyses

Characteristics of patients with malignancy were described for the entire population as well as for the subgroups of delirious and non-delirious patients. Values are depicted as numbers and percentages for categorical variables or median and interquartile range (IQR) for continuous variables. Groups were compared with Fisher’s exact test or Mann–Whitney U test depending on the variable. In unadjusted analyses comparing delirious to non-delirious patients, odds ratios (OR) and 95% confidence intervals (CI) were calculated for in-hospital mortality and for the rate of patients discharged home, and hazard ratios (HR) with 95% CI were computed for ICU and hospital LOS as well as duration of mechanical ventilation with univariate Cox regression with HR < 1 indicating longer length of stay or mechanical ventilation. Regression coefficients and 95% CI were obtained from linear regression for ICU nursing workload as well as ICU and total costs. In multivariate analysis done with multivariate binary logistic regression, multivariate Cox regression and multiple linear regression, the OR, HR and regression coefficients and their 95% CI were adjusted for the following six covariates: presence of sepsis, presence of shock, emergency admission, age, Charlson Comorbidity Index and disease severity (SAPS II). However, in-hospital mortality was only adjusted for presence of sepsis and shock and SAPS II, because the number of events restricted the inclusion of more covariates. We included both sepsis and shock since they only partially overlapped: from 36 patients with shock and 32 patients with sepsis, 16 patients had both. The null hypothesis was rejected with a two-sided *p* value < 0.05. All statistical analyses were performed with IBM SPSS Statistics, version 25, software (IBM, Armonk, NY, USA).

### Ethics statement

This study (PB_2016–01264) was approved by the responsible ethics board of the “Kantonale Ethikkommission des Kanton Zurich” and carried out in accordance with the Declaration of Helsinki, taking into consideration local regulations and standards. On admission to the university hospital, patients signed a general consent, thereby permitting the anonymized use of their non-genetical data for scientific purposes. Due to the retrospective nature of the study and the absence of any experimental intervention, the ethics board waived the need for an additional informed consent.

## Results

### Participants

**Figure 1:**
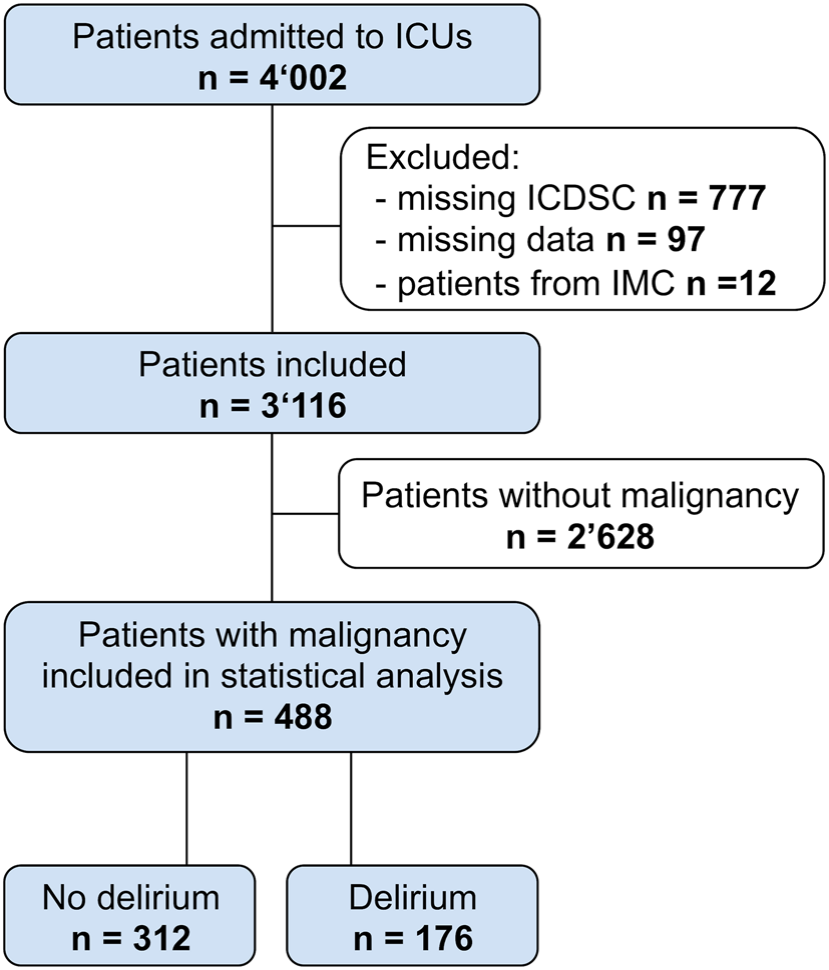
Study flowchart. *ICDSC* intensive care delirium screening checklist, *ICU* intensive care unit, *IMC* intermediate care.

After the initial inclusion of 4002 critically ill patients, 12 patients were excluded due to treatment in intermediate care units, 97 patients due to missing data and 777 patients due to missing ICDSC scores. The latter involved patients who remained comatose or sedated until death and patients treated in one ICU that used preferentially CAM-ICU. Of the remaining 3116 patients, 488 (16%) had malignancy as principal diagnosis (Fig. 1).

### Descriptive data

**Figure 2:**
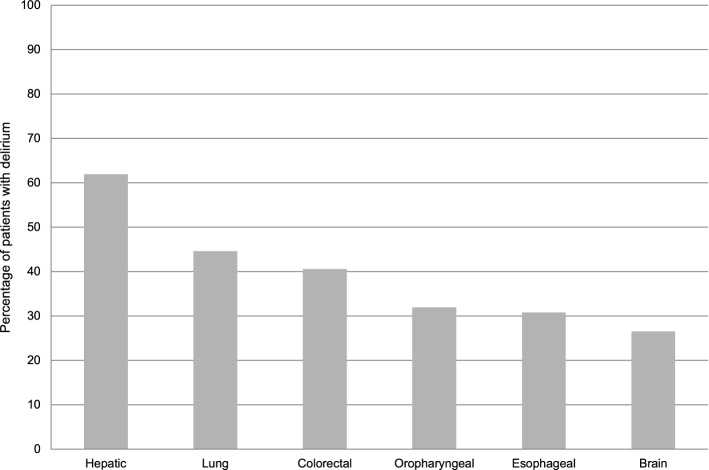
Percentage ICU patients with malignancy who developed ICU delirium stratified by malignancy type. *ICU* intensive care unit.

In this study, 176/488 (36%) patients developed a delirium during their ICU stay. Patient characteristics for patients with malignancy are depicted in Table 1 for the entire population as well as for the subgroups of delirious and non-delirious patients. A comparison of delirium frequencies in patients with malignancy across different types of malignancies is shown in Fig. 2. Comparing delirium frequencies in critically ill oncological patients across common types of care, the following results were obtained: 57% (n = 27/47, 95% CI 43–72%) for thoracic surgery, 40% (n = 54/136; 95% CI 31–48%) for abdominal surgery, 22% (n = 32/145; 95% CI 15–29%) for neurosurgery, and 55% (n = 16/29; 95% CI 37–73%) for internal medicine. Although the Charlson Comorbidity Index was 4 (2–8) for patients with and without delirium, the groups differed significantly (*p* = 0.034). In contrast, no difference in rate of emergency admissions was observed between patients with (51/176 [29%]) and without (81/312 [26%]) delirium (*p* = 0.524). Comparing oncological patients with and without delirium, sepsis occurred in 26/176 (15%) and 6/312 (1.9%) patients (*p* < 0.001), while shock was diagnosed in 30/176 (6.1%) and 6/312 (1.9%) patients, respectively (*p* < 0.001). The SAPS II in delirious and non-delirious patients with malignancy was 41 (27–68) and 24 (17–32), respectively (*p* < 0.001).

**Table 1:** Patient characteristics: Comparison between delirious and non-delirious patients with malignancy.

	All patients	No delirium	Delirium	*p* Value^a^
		(ICDSC < 4)	(ICDSC ≥ 4)	
	n = 488	n = 312	n = 176	
Age (years,) median (IQR)	63 (52–71)	61 (51–69)	66 (55–72)	**0.001**
Male, n (%)	309 (63)	198 (64)	111 (63)	1
**Malignancy type, n (%)**				
Solid malignancy	459 (94)	295 (95)	164 (93)	0.554
Hematological malignancy	29 (5.9)	17 (5)	12 (7)	0.334
**Malignancy, n (%)**				
Brain	98 (20)	72 (23)	26 (15)	**0.034**
Lung	65 (13)	36 (12)	29 (17)	0.129
Oropharyngeal	47 (9.6)	32 (10)	15 (8.5)	0.632
Esophageal	39 (8)	27 (8.7)	12 (6.8)	0.602
Colorectal	37 (7.6)	22 (7.1)	15 (8.5)	0.595
Hepatic	21 (4.3)	8 (2.6)	13 (7.4)	**0.018**
Other	181 (37)	115 (37)	66 (38)	0.922
Metastatic solid tumor, n (%)	190 (39)	125 (40)	65 (37)	0.562
Charlson Comorbidity Index, median (IQR)	4 (2–8)	4 (2–8)	4 (2–8)	**0.034**
Sepsis, n (%)	32 (6.6)	6 (1.9)	26 (15)	** < 0.001**
Shock, n (%)	36 (7.4)	6 (1.9)	30 (6.1)	** < 0.001**
**Residency prior admission, n (%)**				
Home	415 (85)	268 (86)	147 (84)	0.510
Other hospital	58 (12)	36 (12)	22 (13)	0.772
Nursing home	3 (0.6)	1 (0.3)	2 (1.1.)	0.296
Other residency	12 (2.5)	7 (2.2)	5 (2.8)	0.763
Emergency admission, n (%)	132 (27)	81 (26)	51 (29)	0.524
**Type of care, n (%)**				
Neurosurgery	145 (30)	113 (36)	32 (18)	** < 0.001**
Abdominal surgery	136 (28)	82 (26)	54 (31)	0.344
Thoracic surgery	47 (9.6)	20 (6.4)	27 (15)	**0.002**
Otorhinolaryngology/maxillofacial surgery	53 (11)	34 (11)	19 (11)	1
Internal/general medicine	29 (5.9)	13 (4.2)	16 (9.1)	**0.044**
Other service	78 (16)	50 (16)	28 (16)	1
Mechanical ventilation, n (%)	175 (36)	74 (24)	101 (57)	** < 0.001**
SAPS II, median (IQR)	28 (21–43)	24 (17–32)	41 (27–68)	** < 0.001**

*ICDSC* intensive care delirium screening checklist, *IQR* interquartile range, *SAPS II* simplified acute physiology score II.

^a^Comparison of the groups delirium versus no delirium by Fisher's exact or Mann–Whitney U tets.

Bold indicates significance.

### Outcome data

Outcome data for patients with malignancy are provided in Table 2 for the entire population as well as for the subgroups of delirious and non-delirious patients. Adjusted results in Table 3 show that delirium was independently associated with less discharge home, ICU and hospital LOS, duration of mechanical ventilation, ICU nursing workload as well as ICU and total costs. However, delirium was not independently associated with in-hospital mortality in patients with malignancy. For comparison, outcome data is depicted for patients without malignancy and compared between the subgroups of delirious and non-delirious patients (Table 4). As can be seen, delirium does not have a significant influence on outcomes of patients without malignancy.

**Table 2:** Outcome of critically ill patients with malignancy: Comparison between delirious and non-delirious patients.

	All patients	No delirium	Delirium	*p* Value^a^
		(ICDSC < 4)	(ICDSC ≥ 4)	
	n = 488	n = 312	n = 176	
	n (%)	n (%)	n (%)	
In-hospital mortality	39 (8)	9 (2.9)	30 (17)	** < 0.001**
Discharged home	246 (50)	195 (63)	51 (29)	** < 0.001**

	Median (IQR)	Median (IQR)	Median (IQR)	
ICU length of stay (hours)	23 (19–67)	21 (18–25)	81 (25–184)	** < 0.001**
Hospital length of stay (days)	16 (10–24)	14 (8–20)	21 (14–32)	** < 0.001**
Duration of mechanical ventilation (hours)	0 (0–16)	0 (0–0)	16 (0–86)	** < 0.001**

	Median (IQR)	Median (IQR)	Median (IQR)	
Nursing workload (NEMS)	79 (59–218)	72 (54–100)	242 (96–659)	** < 0.001**
ICU costs	4067 (2584–11,222)	2962 (2351–5020)	14,022 (5852–30,784)	** < 0.001**
Total costs	49,750 (32,831–77,523)	40,352 (28,827–60,016)	77,531 (47,074–126,998)	** < 0.001**

*ICDSC* intensive care delirium screening checklist, *ICU* intensive care unit, *IQR* interquartile range, *NEMS* nine equivalents of nursing manpower use score.

^a^Comparison of the groups delirium versus no delirium by Fisher's exact and Mann–Whitney U tests.

Bold indicates significance.

**Table 3:** Outcome of critically ill patients with malignancy: Results from univariate and multivariate analysis.

	n	Univariate analysis^a^	*p* Value	n	Multivariate analysis^b^	*p* Value
		Differences			Differences	
		OR (95% CI)			OR (95% CI)	
In-hospital mortality	488	5.91 (2.87–12.16)	** < 0.001**	488	2.26 (0.93–5.54)	0.074
Discharged home	488	0.46 (0.36–0.59)	** < 0.001**	488	0.37 (0.24–0.57)	** < 0.001**

	n	HR (95% CI)		n	HR (95% CI)	
ICU length of stay (hours)	449^c^	0.25 (0.20–0.32)	** < 0.001**	449^c^	0.30 (0.23–0.37)	** < 0.001**
Hospital length of stay (days)	449^c^	0.45 (0.37–0.55)	** < 0.001**	449^c^	0.62 (0.50–0.77)	** < 0.001**
Duration of mechanical ventilation (hours)	175^d^	0.36 (0.26–0.51)	** < 0.001**	175^d^	0.40 (0.28–0.57)	** < 0.001**

	n	B (95% CI)		n	B (95% CI)	
Nursing workload (NEMS)	488	3.51 (2.97–4.15)	** < 0.001**	485^e^	1.92 (1.67–2.21)	** < 0.001**
ICU costs	488	3.89 (3.29–4.60)	** < 0.001**	485^e^	2.08 (1.81–2.38)	** < 0.001**
Total costs	488	1.96 (1.75–2.20)	** < 0.001**	488^e^	1.44 (1.30–1.60)	** < 0.001**

*B* regression coefficient, *CI* confidence interval, *HR* hazard ratio, *ICU* intensive care unit, *NEMS* nine equivalents of nursing manpower use score, *OR* odds ratio.

^a^Unadjusted differences between the groups delirium vs. no delirium described as OR, HR from univariate Cox regression and regression coefficients from linear regression, each with its 95% confidence intervals.

^b^Adjusted differences between the groups delirium vs. no delirium described as OR from multivariate binary logistic regression, HR from multivariate Cox regression and adjusted regression coefficients from multiple linear regression, each with its 95% confidence intervals. Multivariate models incorporated following covariates: Sepsis, shock, emergency admission, Simplified Acute Physiology Score II (SAPS II), age and Charlson Comorbidity Index ecxcept the multivariate binary logistic regression of in-hospital mortality which incorporated only sepsis, shock and SAPS II as covariates. Due to nonproportionality in multivariate Cox regression, SAPS II was entered as time-varying covariate in the analysis of ICU and duration of mechanical ventilation, and SAPS II and age were entered as time-varying covariates in the analysis of hospital length of stay. Due to skewedness of ICU nursing workload and ICU and total cost data, the natural log transformation was performed in both linear and multiple linear regression.

^c^Patients with in-hospital death excluded (n = 39).

^d^Only patients with mechanical ventilation > 0 h included due to natural log transformation.

^e^Patients with SAPS II = 0 lost due to natural log transformation of SAPS II (n = 3).

HR < 1 indicates longer length of stay and longer mechanical ventilation.

Bold indicates significance.

**Table 4:** Outcome of critically ill patients without malignancy: Comparison between delirious and non-delirious patients.

	All patients	No delirium	Delirium	*p* Value^a^	Univariate analysis^b^	*p* Value
		(ICDSC < 4)	(ICDSC ≥ 4)		Differences	
	n = 2628	n = 1536	n = 1092			
	n (%)	n (%)	n (%)		OR (95% CI)	
In-hospital mortality	240 (9.1)	152 (9.9)	88 (8.0)	0.114	1.190 (0,919–1.541)	0.114
Discharged home	1016 (38)	591 (38)	425 (39)	0.871	0.913 (0.818–1.019)	0.871

	Median (IQR)	Median (IQR)	Median (IQR)		HR (95% CI)	
ICU length of stay (hours)	38 (20–1111)	37 (21–107)	39 (20–118)	0.694	0.980 (0.904–1.063)	0.626
Hospital length of stay (days)	12 (7–21)	12 (7–20)	12 (7–21)	0.686	0.956 (0.881–1.037)	0.276
Duration of mechanical ventilation (hours)	8 (0–40)	8 (0–32)	8 (0–40)	0.802	0.975 (0.881–1.080)	0.632

	Median (IQR)	Median (IQR)	Median (IQR)		B (95% CI)	
Nursing workload (NEMS)	127 (72–390)	127 (72–384)	127 (72–414)	0.950	0.023 (− 0.069–0.115)	0.622
ICU costs	6427 (3113–18,247)	6664 (3149–17,817)	6122 (3073–19,382)	0.807	0.018 (− 0.074–0.111)	0.701
Total costs	45276 (28,583–80,076)	46,172 (29,502–78,424)	43,919 (27,541–82,925)	0.600	− 0.004 (− 0.067–0.060)	0.904

*B* regression coefficient, *CI* confidence interval, *HR* hazard ratio, *ICDSC* intensive care delirium screening checklist, *ICU* intensive care unit, *IQR* interquartile range, *NEMS* nine equivalents of nursing manpower use score, *OR* odds ratio.

^a^Comparison of the groups delirium vs. no delirium by Fisher's exact and Mann–Whitney U tests.

^b^Unadjusted differences between the groups delirium vs. no delirium described as OR, HR from univariate Cox regression and regression coefficients from linear regression after natural log transformation for NEMS and costs, each with its 95% confidence intervals.

## Discussion

In this large sample analysis of 488 critically ill patients with malignancies treated in a University Hospital, 36% developed delirium in the ICU. Compared to non-delirious patients with malignancy, oncological patients with delirium were older, had a higher comorbidity burden, were more severely ill, were more frequently mechanically ventilated and experienced more often sepsis and shock. Delirium showed high frequencies in patients with hepatic, lung and colorectal malignancies. In addition, it was particularly frequent in patients from thoracic and abdominal surgery while it developed only in one out of five neurosurgical patients. Delirium in patients with malignancy was independently associated with lower odds to be discharged home, longer ICU and hospital LOS, longer duration of mechanical ventilation, increased ICU nursing workload as well as higher ICU and total costs. Whereas delirium was a strong marker of in-hospital mortality, multivariate analysis revealed that it was not independently associated with in-hospital mortality in this population.

More than one out of three oncological ICU patients developed delirium during their ICU stay. This frequency of delirium in this special population of critically but not terminally ill oncological ICU patients lies between those reported for general wards and palliative care units and is comparable to prevalence rates registered in general ICU populations which range from 19 to 82%^1,3–5,9^. Very limited and controversial data have been available in this particular patient population so far^10,11,27^. The much higher delirium frequency of 95% observed by Almeida et al. (n = 170) might be explained by three reasons: (1) by the divergent population of severely ill, mechanically ventilated patients; (2) by preventive measures including delirium monitoring, daily awakening trials and early mobilization implemented at the study hospital; (3) by patients in persistent coma (for example due to non-convulsive status epilepticus which can occur in critically ill cancer patients^28^) or sedation until death without delirium screening potentially influencing the frequency observed in the present study^10^. The lower frequency of 23% reported by Sánchez-Hurtado et al.^11^ (n = 109) might derive from divergent baseline characteristics and screenings. Despite demonstration of a higher sensitivity and specificity with the CAM-ICU (80% and 96%, respectively) than with the ICDSC (74% and 82%, respectively) for the diagnosis of delirium^5^, since screening with the CAM-ICU was done only once daily compared to ICDSC performed three times daily in the present study, the study by Sánchez-Hurtado et al. might have missed delirium in some cases due to its highly fluctuating course^17^. Gouveia et al.^27^ (n = 135) observed a similar delirium frequency of 39% to the one reported in the present study.

Hepatic and colorectal as well as lung cancer patients often developed delirium while delirium was less frequent in brain cancer patients. Studies on general ward and ICU patients who had undergone oncological surgery reported the following delirium frequencies: 7% for primary pulmonary malignancy^29^, 8% for hepatocellular carcinoma^30^, 7% for glioblastoma^31^ and 14% for colorectal carcinoma^32^. Due to the selection of ICU patients, delirium was more frequent in the present study. To the best of our knowledge, this study is the first to report ICU delirium frequencies on different malignancy types. Although the small sample sizes of subgroups limited our study’s results on delirium frequency in malignancy types, this study reports high frequencies of ICU delirium and suggests the presence of differences in frequency of delirium between malignancy types. However, due to the retrospective nature of the present study, no causal relationship between malignancy and delirium as for example in hepatic encephalopathy can be established. Further studies are needed to address this issue.

Delirium frequencies among medical ICU patients and ICU patients from thoracic and abdominal surgery were high while neurosurgical ICU patients developed delirium less often. Compared with previous studies on postoperative delirium which showed incidence rates from 13 to 50%, the presented results for surgical patients are rather high^33^. Possible reasons include the more severely ill ICU population and the inclusion of patients with pre-existing neurocognitive disorders. The delirium frequency in the medical ICU lies between prior reports from van den Boogaard et al. and Ely et al. who found incidence rates of 40% in general medical ICU patients and 82% in exclusively mechanically ventilated medical ICU patients, respectively^1,34^. The results on neurosurgical ICU patients are surprising and might be explained by patient selection. However, they are supported by previous publications by van den Boogaard et al. and Wang et al. which registered delirium frequencies of 10% and 20%, respectively^34,35^.

Resource utilization with respect to ICU and hospital LOS as well as ICU nursing workload and duration of mechanical ventilation was increased in delirious patients with malignancy when compared to non-delirious patients. Accordingly, also ICU and total costs per case were increased. Whereas delirium in oncological ICU patients has been associated with longer ICU and hospital LOS and duration of mechanical ventilation by Sánchez-Hurtado et al.^11^, Almeida et al. did not find any significant association^10^. However, the latter study was significantly underpowered for the comparison of patients with and without delirium. While the findings presented in this study are consistent with previous publications on general ICU populations^1,6,7^ and while longer hospital LOS has been reported in delirious palliative care unit patients^36^, this large study adds new data on resources utilized by delirious ICU patients with malignancy. Since ICU LOS and duration of mechanical ventilation are related to complications such as nosocomial infections and ventilator related lung injury, these results have relevant implications for clinical routine. The median ICU costs for patients with malignancy increasing fivefold with delirium are in proportion to the median ICU length of stay which quadrupled with delirium. The higher nursing workload augmented costs additionally. Due to an increasing number of oncological patients admitted to the ICU^13^, the higher costs caused by oncological ICU patients with delirium have important implications on health care systems.

In the present study, while being a strong marker of higher in-hospital mortality, delirium was not independently associated with in-hospital mortality in critically ill patients with malignancy. Although this supports findings from Sanchez-Hurtado et al.^11^, it contradicts results from unadjusted analysis published by Almeida et al. and Praça et al.^10,37^. However, while Almeida et al. reported lack of power for the comparison of delirious and non-delirious patients, Praça et al. studied a terminally ill population discharged from the ICU after a decision to withhold life-sustaining therapies. Whereas two of the three studies from palliative care units reporting adjusted results did not observe an independent relationship between delirium and in-hospital mortality^38,39^, Shin et al.^40^ showed an independent association between delirium and in-hospital mortality (OR [95% CI] 0.394 [0.244–0.635], *p* = 0.0003). Our data suggest that delirium is not independently associated with short-term mortality in critically ill oncological patients, as has been previously shown by Klein Klouwenberg et al.^41^ for medical and surgical ICU patients. Thus, delirium probably constitutes rather a marker for illness severity than being independently related to increased in-hospital mortality. This interpretation is also supported by the observation that patients with delirium had significantly higher SAPS II scores, were more often mechanically ventilated and suffered more frequently from sepsis and shock than patients without delirium.

Delirium had no significant effect on resource utilization and in-hospital mortality in unadjusted analyses of patients without malignancy. In-hospital mortality and other outcome parameters in patients with and without malignancy were similar. More work on the population of ICU patients without malignancy will be published in a future study.

Delirium should be met with systematic delirium management including preventive measures, early recognition and therapy, since different interventions have been demonstrated to reduce delirium frequency in general ICU studies^42–44^. Future studies should address the association between delirium interventions and outcomes including long-term adverse events.

This study is limited by its retrospective and observational design. While this enabled a larger sample size providing a representative overview of delirium in critically ill oncological patients, the use of pre-existing data led to several noteworthy limitations: (1) it impeded management of delirium as a time-dependent variable in multivariate analysis for which time-varying variables would have been needed. This restricted generalizability and causal inferences. (2) It limited the choice of variables included. Therefore, neither delirium treatment nor long-term consequences such as cognitive impairment, psychopathologies, impact on quality of life and long-term mortality can be addressed by the present study. In addition, information on oncological treatment was not collected. (3) We had to deal with a considerable number of missing ICDSC data in the medical ICU since this particular ICU screened patients primarily with the CAM-ICU, which may have led to an underrepresentation of medical ICU patients due to the exclusion of patients with CAM-ICU screening, impeding generalizability of results concerning this particular subgroup. Despite the high number of patients, the inclusion of more variables in the multivariate statistics of in-hospital mortality was restricted by the small number of deaths since approximately ten events are required for every variable included. In this study, delirium diagnosis was defined with the ICDSC at a cut-off score ≥ 4. We showed in a previously published study that the ICDSC had a higher sensitivity and was more accurate as screening tool compared to the CAM-ICU^17^. Nevertheless, the chosen cut-off score of the ICDSC could have resulted in an overestimation of the true delirium frequency. ABCDE bundle integration was not a particular focus during the study period at our institution. Future studies should describe this in more detail according to the published PAD/PADIS guidelines^45^.

In conclusion, delirium occurred in 36% of critically ill oncological patients. Delirious and non-delirious patients differed in age, comorbidity burden, illness severity and frequency of sepsis and shock. Delirium in patients with malignancy was independently associated with lower odds to be discharged home, longer ICU and hospital LOS, longer duration of mechanical ventilation, increased ICU nursing workload as well as higher ICU and total costs. Although delirium was a strong marker of in-hospital mortality, it was not independently associated with in-hospital mortality in multivariate analysis. This suggests that delirium might rather be a marker for severe illness but not than an independent risk factor of in-hospital mortality. Future studies should address the association between delirium and long-term outcomes in critically ill patients with malignancies.

## Supplementary Information

Supplementary Information.

## Data Availability

The datasets analyzed during the current study are available from the corresponding author on reasonable request.

## References

[1] Ely EW (2004). Delirium as a predictor of mortality in mechanically ventilated patients in the intensive care unit. JAMA.

[2] Pandharipande P (2007). Motoric subtypes of delirium in mechanically ventilated surgical and trauma intensive care unit patients. Intensive Care Med..

[3] Veiga D (2012). Postoperative delirium in intensive care patients: Risk factors and outcome. Rev. Bras. Anestesiol..

[4] Rudiger A (2016). Intra-operative events during cardiac surgery are risk factors for the development of delirium in the ICU. Crit. Care.

[5] Wilson JE (2020). Delirium. Nat. Rev. Dis. Primers.

[6] Salluh JI (2015). Outcome of delirium in critically ill patients: Systematic review and meta-analysis. BMJ.

[7] Milbrandt EB (2004). Costs associated with delirium in mechanically ventilated patients. Crit. Care Med..

[8] Pandharipande PP (2013). Long-term cognitive impairment after critical illness. N. Engl. J. Med..

[9] Bush SH (2018). Delirium in adult cancer patients: ESMO Clinical Practice Guidelines. Ann. Oncol..

[10] Almeida IC (2014). The impact of acute brain dysfunction in the outcomes of mechanically ventilated cancer patients. PLoS ONE.

[11] Sánchez-Hurtado LA (2018). Incidence of delirium in critically Ill cancer patients. Pain Res. Manag..

[12] Lawlor PG, Bush SH (2015). Delirium in patients with cancer: Assessment, impact, mechanisms and management. Nat. Rev. Clin. Oncol..

[13] Shimabukuro-Vornhagen A, Boll B, Kochanek M, Azoulay E, von Bergwelt-Baildon MS (2016). Critical care of patients with cancer. CA Cancer J. Clin..

[14] Schubert M (2018). A hospital-wide evaluation of delirium prevalence and outcomes in acute care patients—A cohort study. BMC Health Serv. Res..

[15] Sessler CN (2002). The Richmond Agitation-Sedation Scale: Validity and reliability in adult intensive care unit patients. Am. J. Respir. Crit. Care Med..

[16] Bergeron N, Dubois MJ, Dumont M, Dial S, Skrobik Y (2001). Intensive Care Delirium Screening Checklist: Evaluation of a new screening tool. Intensive Care Med..

[17] Boettger S (2017). Delirium in the intensive care setting: A reevaluation of the validity of the CAM-ICU and ICDSC versus the DSM-IV-TR in determining a diagnosis of delirium as part of the daily clinical routine. Palliat. Support Care.

[18] Gusmao-Flores D, Salluh JI, Chalhub R, Quarantini LC (2012). The confusion assessment method for the intensive care unit (CAM-ICU) and intensive care delirium screening checklist (ICDSC) for the diagnosis of delirium: A systematic review and meta-analysis of clinical studies. Crit. Care.

[19] Boettger S (2018). Screening for delirium with the Intensive Care Delirium Screening Checklist (ICDSC): A re-evaluation of the threshold for delirium. Swiss Med. Wkly.

[20] Boettger S (2019). Screening for delirium with the Intensive Care Delirium Screening Checklist (ICDSC): Symptom profile and utility of individual items in the identification of delirium dependent on the level of sedation. Palliat. Support Care.

[21] Boettger S (2017). Delirium in the intensive care setting and the Richmond Agitation and Sedation Scale (RASS): Drowsiness increases the risk and is subthreshold for delirium. J. Psychosom. Res..

[22] Reis Miranda D, Moreno R, Iapichino G (1997). Nine equivalents of nursing manpower use score (NEMS). Intensive Care Med..

[23] Quan H (2011). Updating and validating the Charlson comorbidity index and score for risk adjustment in hospital discharge abstracts using data from 6 countries. Am. J. Epidemiol..

[24] Le Gall JR, Lemeshow S, Saulnier F (1993). A new Simplified Acute Physiology Score (SAPS II) based on a European/North American multicenter study. JAMA.

[25] BFS. 1-43 + Anhang (Bundesamt für Statistik, Abteilung für Bevölkerung und Beschäftigung, Neuchâtel, 1997).

[26] SGI-SSMI. 1-30 (Basel, 2013).

[27] Gouveia BR, Jomar RT, Valente TCO (2019). Delirium in cancer patients admitted to the intensive care unit: A retrospective study. Rev. Bras. Ter. Intensiva.

[28] Gutierrez C, Chen M, Feng L, Tummala S (2019). Non-convulsive seizures in the encephalopathic critically ill cancer patient does not necessarily portend a poor prognosis. J. Intensive Care.

[29] Hayashi K, Motoishi M, Sawai S, Horimoto K, Hanaoka J (2019). Postoperative delirium after lung resection for primary lung cancer: Risk factors, risk scoring system, and prognosis. PLoS ONE.

[30] Chen YL (2015). Low hemoglobin level is associated with the development of delirium after hepatectomy for hepatocellular carcinoma patients. PLoS ONE.

[31] Flanigan PM (2018). Postoperative delirium in glioblastoma patients: Risk factors and prognostic implications. Neurosurgery.

[32] Yang Z (2020). Prevalence and risk factors for postoperative delirium in patients with colorectal carcinoma: A systematic review and meta-analysis. Int. J. Colorectal Dis..

[33] Inouye SK, Westendorp RG, Saczynski JS (2014). Delirium in elderly people. Lancet.

[34] van den Boogaard M, Schoonhoven L, van der Hoeven JG, van Achterberg T, Pickkers P (2012). Incidence and short-term consequences of delirium in critically ill patients: A prospective observational cohort study. Int. J. Nurs. Stud..

[35] Wang CM (2020). Incidence and risk factors of postoperative delirium in patients admitted to the ICU after elective intracranial surgery: A prospective cohort study. Eur. J. Anaesthesiol..

[36] Mercadante S, Adile C, Ferrera P, Cortegiani A, Casuccio A (2017). Delirium assessed by Memorial Delirium Assessment Scale in advanced cancer patients admitted to an acute palliative/supportive care unit. Curr. Med. Res. Opin..

[37] Praça APA, Nassar AP, Caruso P (2019). Outcomes of cancer patients discharged from ICU after a decision to forgo life-sustaining therapies. Crit. Care Med..

[38] Mori M (2011). Changes in symptoms and inpatient mortality: A study in advanced cancer patients admitted to an acute palliative care unit in a comprehensive cancer center. J. Palliat. Med..

[39] Tarumi Y (2011). Evaluation of the Palliative Prognostic Score (PaP) and routinely collected clinical data in prognostication of survival for patients referred to a palliative care consultation service in an acute care hospital. J. Pain Symptom Manag..

[40] Shin SH (2014). Characteristics and outcomes of patients admitted to the acute palliative care unit from the emergency center. J. Pain Symptom Manag..

[41] Klein Klouwenberg PM (2014). The attributable mortality of delirium in critically ill patients: prospective cohort study. BMJ.

[42] Pandharipande PP (2007). Effect of sedation with dexmedetomidine vs lorazepam on acute brain dysfunction in mechanically ventilated patients: The MENDS randomized controlled trial. JAMA.

[43] Riker RR (2009). Dexmedetomidine vs midazolam for sedation of critically ill patients: A randomized trial. JAMA.

[44] Schweickert WD (2009). Early physical and occupational therapy in mechanically ventilated, critically ill patients: A randomised controlled trial. Lancet.

[45] Devlin JW (2018). Clinical practice guidelines for the prevention and management of pain, agitation/sedation, delirium, immobility, and sleep disruption in adult patients in the ICU. Crit. Care Med..

