# Supplementary material for: Outcome, demography and resource utilization in ICU Patients with delirium and malignancy
Source: Sci Rep. 2021 Sep 21;11:18756. doi: 10.1038/s41598-021-98200-8 (PMC8455636; doi:10.1038/s41598-021-98200-8)
Supplement: Supplementary file 1 — Supplementary Information. [file 41598_2021_98200_MOESM1_ESM.pdf]

Outcome, demography and resource utilization in ICU Patients with delirium and malignancy

Authors

Mattia Sieber[1°], Alain Rudiger[2°], Reto Schüpbach[3], Bernard Krüger[4], Maria Schubert[5+], Dominique Bettex[4*+]

* corresponding author

° equally contributing first authors

+ equally contributing last authors

Affiliations

[1]Department of Internal Medicine, Zuger Kantonsspital, Landhausstrasse 11, CH-6340 Baar, Switzerland

[2]Department of Internal Medicine, Limmattalspital Zurich, Urdorferstrasse 100, CH-8952 Schlieren, Switzerland

[3]Institute of Intensive Care, University Hospital Zurich and University of Zurich, Rämistrasse 100, CH-8091 Zurich, Switzerland.

[4*]Cardio-Surgical Intensive Care Unit, Institute of Anesthesiology, University Hospital Zurich and University of Zurich, Rämistrasse 100, CH-8091 Zurich, Switzerland.

[5]School of Health Professions, Institute of Nursing, Zurich University of Applied Science, Technikumstr. 81, P.O. Box, 8401, Winterthur, Switzerland

Corresponding author

Prof. Dr. med. Dominique Bettex

Institute of Anesthesiology

University Hospital Zurich

Rämistrasse 100

CH – 8091 Zurich

Switzerland

Email: dominique.bettex@usz.ch

Phone: +41 44 255 11 11

Supplementary Table 1. Patient characteristics: Comparison between patients with malignancy with and without existing ICDSC score

	**ICDSC absent** *n = 83*	**ICDSC present** *n = 488*	***p* value[a]**
Age (years,) median (IQR)	64 (54-72)	63 (52-71)	0.594
Male, n (%)	54 (65)	309 (63)	0.806
Malignancy type, n (%)			
Solid malignancy	64 (77)	459 (94)	**<0.001**
Hematological malignancy	19 (23)	29 (6)	**<0.001**
Malignancy, n (%)			
Brain	18 (22)	98 (20)	0.768
Lung	8 (10)	65 (13)	0.476
Oropharyngeal	9 (11)	47 (10)	0.692
Esophageal	3 (3.6)	39 (8)	0.252
Colorectal	2 (2.4)	37 (7.6)	0.1
Hepatic	2 (2.4)	21 (4.3)	0.557
Other	41 (49)	181 (37)	0.038
Metastatic solid tumor, n (%)	34 (41)	190 (39)	0.717
Charlson Comorbidity Index, median (IQR)	4 (2-8)	4 (2-8)	0.836
Sepsis, n (%)	11 (13)	32 (6.6)	**0.042**
Shock, n (%)	6 (7.2)	36 (7.4)	1
Emergency admission, n (%)	34 (41)	132 (27)	**0.013**
Type of care, n (%)			
Neurosurgery	20 (24)	145 (30)	0.359
Abdominal surgery	11 (13)	136 (28)	**0.004**
Thoracic surgery	0 (0)	47 (9.6)	**0.001**
Otorhinolaryngology / maxiollofacial surgery	11 (13)	53 (11)	0.571
Internal / general medicine	25 (30)	29 (5.9)	**<0.001**
Other service	16 (19)	78 (16)	0.428
SAPS II, median (IQR)	40 (24-54)	28 (21-43)	**0.001**

ICDSC Intensive Care Delirium Screening Checklist, IQR Interquartile Range, SAPS II Simplified Acute Physiology Score II

[a] *Comparison of the groups delirium vs. no delirium by Fisher's exact or Mann-Whitney U tets*

Bold indicates significance
